# Minimizing Cholesterol-Induced Aggregation of Membrane-Interacting DNA Origami Nanostructures

**DOI:** 10.3390/membranes11120950

**Published:** 2021-11-30

**Authors:** Jasleen Kaur Daljit Singh, Minh Tri Luu, Jonathan F. Berengut, Ali Abbas, Matthew A. B. Baker, Shelley F. J. Wickham

**Affiliations:** 1 School of Chemistry, University of Sydney, Sydney, NSW 2006, Australia; jasleen.daljitsingh@sydney.edu.au (J.K.D.S.); m.luu@sydney.edu.au (M.T.L.); jonathan.berengut@sydney.edu.au (J.F.B.); 2 School of Chemical and Biomolecular Engineering, University of Sydney, Sydney, NSW 2006, Australia; ali.abbas@sydney.edu.au; 3 The University of Sydney Nano Institute, University of Sydney, Sydney, NSW 2006, Australia; 4 School of Biotechnology and Biomolecular Sciences, University of New South Wales, Sydney, NSW 2052, Australia; matthew.baker@unsw.edu.au; 5 CSIRO Synthetic Biology Future Science Platform, GPO Box 2583, Brisbane, QLD 4001, Australia; 6 School of Physics, University of Sydney, Sydney, NSW 2006, Australia

**Keywords:** DNA origami, DNA nanotechnology, cholesterol, aggregation

## Abstract

DNA nanotechnology provides methods for building custom membrane-interacting nanostructures with diverse functions, such as shaping membranes, tethering defined numbers of membrane proteins, and transmembrane nanopores. The modification of DNA nanostructures with hydrophobic groups, such as cholesterol, is required to facilitate membrane interactions. However, cholesterol-induced aggregation of DNA origami nanostructures remains a challenge. Aggregation can result in reduced assembly yield, defective structures, and the inhibition of membrane interaction. Here, we quantify the assembly yield of two cholesterol-modified DNA origami nanostructures: a 2D DNA origami tile (DOT) and a 3D DNA origami barrel (DOB), by gel electrophoresis. We found that the DOT assembly yield (relative to the no cholesterol control) could be maximised by reducing the number of cholesterols from 6 to 1 (2 ± 0.2% to 100 ± 2%), optimising the separation between adjacent cholesterols (64 ± 26% to 78 ± 30%), decreasing spacer length (38 ± 20% to 95 ± 5%), and using protective ssDNA 10T overhangs (38 ± 20% to 87 ± 6%). Two-step folding protocols for the DOB, where cholesterol strands are added in a second step, did not improve the yield. Detergent improved the yield of distal cholesterol configurations (26 ± 22% to 92 ± 12%), but samples re-aggregated after detergent removal (74 ± 3%). Finally, we confirmed functional membrane binding of the cholesterol-modified nanostructures. These findings provide fundamental guidelines to reducing the cholesterol-induced aggregation of membrane-interacting 2D and 3D DNA origami nanostructures, improving the yield of well-formed structures to facilitate future applications in nanomedicine and biophysics.

## 1. Introduction

The development of the DNA origami [1] technique has led to rapid developments in the field of DNA nanotechnology. Over the past 15 years, complex 2D and 3D DNA origami nanostructures have been created [2] as well as dynamic DNA nanostructures that respond to environmental and external triggers [3]. The addressability provided by the DNA origami technique allows for the precise placement of functional molecules such as proteins [4], nanoparticles [5,6] and hydrophobic groups [7] on DNA nanostructures. The nanoscale precision and biocompatibility of DNA nanostructures has resulted in a growing interest in the use of DNA nanostructures for biomimetic applications [8,9] such as mimicking transmembrane proteins [10,11,12] and membrane-interacting proteins [13,14,15]. Typically, DNA nanostructures are functionalized with hydrophobic groups, such as cholesterols [7,10,16,17,18,19,20], tocopherol [21], alkyl chains [22], or porphyrins [11], to facilitate interaction with membranes. Of these hydrophobic groups, cholesterol groups with TEG linkers are most commonly used [7,10,16,17,18,19,20] as they are commercially available and their membrane interaction is well characterized [23,24]. Cholesterol-labelled DNA origami nanostructures have been used extensively for membrane labelling [25,26] and membrane shaping [4,18], as well as the formation of transmembrane nanopores [7,16] that allow for molecular transport across the membrane.

Despite their extensive use, there exists a gap in the systematic study of the aggregation of cholesterol-labelled DNA origami nanostructures. Cholesterol-modified DNA nanostructures have been shown to aggregate and form higher-order structures [14,19,27,28]. This aggregation limits the yield of successfully assembled nanostructures [29], can result in deformation of structures [14], and can reduce membrane binding yield [28]. For example, the addition of a 5T spacer and an increase in cholesterol number from 4 to 8 has been shown to promote aggregation and decrease the membrane binding of wireframe DNA cubes [28]. Increasing the number of cholesterols on a rectangular DNA origami tile has been shown to increase the yield of dimer formation from <5% (no cholesterols) to ~70% (14 to 18 cholesterols), with a further increase in cholesterols resulting in deformation and folding of the structure [14]. The functionalization of 3D DNA origami nanopores with 24 cholesterol groups resulted in a smeared band on agarose gels, indicating aggregation, unless supplemented with surfactants such as SDS [30]. Recently, it has been shown that the yield of 4-helix bundle monomers decreased from 83% to between 35 and 77% with the addition of just one cholesterol, with the position of the cholesterol affecting the yield [29]. The number and spacing of cholesterol groups have also been shown to have a large effect on membrane binding [31,32] and cellular uptake [33,34] of DNA nanostructures. However, the effect of varying these parameters on the aggregation of cholesterol-modified DNA origami nanostructures is yet to be systematically evaluated. 

Several strategies have been proposed to minimize cholesterol-mediated aggregation of DNA nanostructures. It has been shown that placing a single-stranded DNA (ssDNA) poly-thymine overhang next to a cholesterol group can minimise the aggregation of cholesterol-modified DNA strands [29]. This strategy also worked for a small (<200 bp) 4-helix bundle DNA nanopore with four cholesterol groups [29] but has not been tested for larger DNA origami nanostructures (>7000 bp), which typically have higher numbers of hydrophobic groups, up to 24–26 [7,21,30]. Other methods to minimise aggregation include the use of surfactants [14,27], and a two-step folding process where structures are folded with only unmodified DNA strands in the first step, followed by the addition of cholesterol-modified DNA strands in the second step [7,16]. However, surfactants often require downstream removal steps for in vivo applications [35], and two-step assembly adds complexity to the process. The problem of cholesterol-mediated aggregation of DNA nanostructures has also been circumvented by labelling the membrane directly with cholesterol-modified ssDNA, and then adding DNA nanostructures containing complementary ssDNA overhangs [34]. This has been shown to be an effective strategy to overcome aggregation but requires the ability to label target membranes with cholesterol-DNA prior to adding DNA nanostructures. For many applications, such as in vivo use, this may not be possible. 

In this work, we focus on optimising the design of cholesterol-labelled DNA origami nanostructures to minimise aggregation, by measuring the monomer folding yield. We test the aggregation of two different cholesterol-modified DNA origami nanostructures: DNA origami tile (DOT) [1] and DNA origami barrel (DOB) [36]. The DOT is a flexible single-layered 24-helix rectangular 2D DNA nanostructure (60 × 90 nm), while the DOB is a barrel-shaped rigid 3D structure (52 × 30 nm). In a previous study, we showed that increasing the number of cholesterols does not always increase the membrane binding, and that the optimum number of cholesterols for the DOT structure is 4–8 [37]. Here, we compare the aggregation for two different configurations of cholesterols on the DOT: a four-cholesterol rectangular (R) configuration and a six-cholesterol circular (C) configuration. We use six instead of eight cholesterols as it allows for even spacing between the cholesterol groups on the DOT. We vary the separation between the cholesterol groups (20 nm to 60 nm diameter), the spacer length connecting the cholesterols to the DOT (1.4–15.2 nm), and the type of overhang used (10-nt or 10-T). Next, we evaluate the effect of spacer length, overhang and folding protocol (one- or two-step and surfactant) on the aggregation of the DOB. We then compare the effect of varying the number of cholesterols (1–6) for DOT and DOB. For both the DOT and DOB, aggregation is determined by quantifying the nanostructure assembly yield using agarose gel electrophoresis. A high yield indicates low aggregation, and a low yield indicates high aggregation. Finally, for the design with the highest yield, we compare the liposome binding of the DOT and the DOB using a gel-shift assay and transmission electron microscopy.

## 2. Materials and Methods

List of reagents used is given in Table S1.

### 2.1. The Design and Folding of DNA Origami Nanostructures

**Figure 1:**
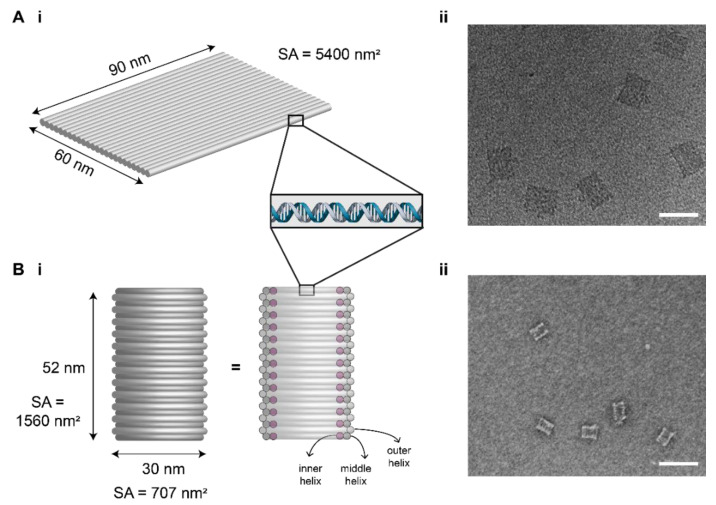
Design of DNA origami nanostructures used in this study. (**A**) DNA origami tile (DOT); (**B**) DNA origami barrel (DOB). (**i**) Schematic of the nanostructures. (**ii**) TEM images of nanostructures. Scale bar: 100 nm. SA = surface area.

Two DNA origami nanostructures were used: DNA origami tile (DOT) and DNA origami barrel (DOB). The design of the DOT was adapted from a previous study [1] and was modified using Picasso software [38]. The DOT is a flexible 2D nanostructure made of 24 parallel DNA helices and dimensions of approximately 60 nm × 90 nm × 2 nm (Figure 1A(i)).

The DOB design was also adapted from a previous study [36] and modified using caDNAno [39]. The DOB is a rigid 3D structure made of three layers of DNA helices (inner layer, middle layer, outer layer) and has dimensions of approximately 52 nm × 30 nm (Figure 1B(i)). 

Both the DOT and the DOB were folded in folding buffer (5 mM Tris, 1 mM EDTA, 12 mM MgCl_2_, pH 8.0) using 10 nM of scaffold (M13mp18 ssDNA) and 10× excess (100 nM) of DNA staple strands. For cholesterol attachment, 21-nt ssDNA handles were extended from the DOT/DOB, which could hybridise to complementary cholesterol-modified ssDNA strands. A 2× excess of cholesterol-modified ssDNA relative to the concentration of handle staples (e.g., for 6 cholesterols in circular configuration, cholesterol strand = 100 nM × 6 × 2 = 1200 nM) was added during folding. The DOT was annealed over 3 h (80 °C for 15 min, then 60–4 °C in 56 steps over 3 h) while the DOB was annealed over 18 h (65 °C for 15 min, then 50–40 °C in 10 steps over 18 h). 

For the folding of DOB with detergent, 1% octyl β-D-glucopyranoside (OG) was added to the folding mix. For two-step folding of the DOB, the DOB was folded without the cholesterol-modified strands. Upon folding and purification by PEG-precipitation, cholesterol-modified strands (with/without 1% OG) were pre-heated for 1 h at 37 °C and then added to the sample at 20× excess to handles (1200 µM) and incubated for 1 h at 37 °C. To remove the detergent, 40 µL sample was dialysed overnight in 1 L of folding buffer using the Slide-A-Lyzer MINI Dialysis Device (10K MWCO, 0.1 mL, Life Technologies, Carlsbad, CA, USA).

Successful assembly of the DOT and DOB was verified using agarose gel electrophoresis and transmission electron microscopy (Figure 1A(ii),B(ii)). The absolute yield of the DOT band as a percentage of the scaffold DNA added was determined by evaluating the DOT band intensity as a percentage of the total lane intensity above the band (Figure S1). The normalized yield was calculated by normalizing the yield in each lane to the control (no cholesterol) lane. Two repeats of folding and gel analysis were conducted, and the yields were averaged across the two repeats, with error calculated by determining the standard deviation across the repeats. For statistical analysis, unpaired *t*-tests were performed.

### 2.2. PEG-Precipitation of DOB

For the two-step cholesterol addition protocol with the DOB, the DOB was purified by PEG precipitation to remove excess staple strands [40]. Briefly, 600 µL of folding buffer was added to 100 µL of the folded DOB and the MgCl_2_ concentration was adjusted to 20 mM. Then, 700 µL of PEG buffer (15% PEG 8000 (*w*/*v*), 5 mM Tris, 1 mM EDTA, 505 mM NaCl) was added and the solution was centrifuged at 18,000× *g* at RT for 25 min. The supernatant was removed using a pipette and discarded, then the pellet was air dried and dissolved in 100 µL of folding buffer.

### 2.3. Purification of DNA Origami Nanostructures for Membrane Binding Experiments

Agarose gel electrophoresis [41] was used to purify the DOTs and DOBs. The samples were loaded onto a 2% agarose gel (in 0.5× TBE, supplemented with 11 mM MgCl_2_ and SyBr Safe DNA gel stain) run at 60 V for 2.5 h at RT. After electrophoresis, the gels were viewed under an LED Blue Light Transilluminator (Fisher Biotec, Wembley, WA, Australia) and the DOT and DOB bands were excised. The extracted bands were transferred to Freeze ‘N Squeeze DNA Gel Extraction spin columns (Bio-Rad) and centrifuged for 10 min at 18,000× *g* and 4 °C. For subsequent membrane binding experiments, the concentration of the purified DOT and DOB was determined using a Nanodrop (Thermo Fisher Scientific, Waltham, MA, USA) to measure absorbance at 260 nm. The solutions were stored at 4 °C.

### 2.4. Formation of Small Unilamellar Vesicles (SUVs)

Lipid mixture of 49.9% DOPE (1,2-dioleoyl-sn-glycero-3-phosphoethanolamine), 49.9% DOPC (1, 2-dioleoyl-sn-glycero-3-phosphocholin) and 0.1% PE-biotin (1,2-dioleoyl-sn-glycero-3-phosphoethanolamine-N-biotinyl) was prepared at 10 mg/mL in chloroform and stored at −20 °C. For SUV preparation, 1 mL of the lipid mixture was transferred to a round-bottom glass tube and the mixture was dried under nitrogen gas for 20 min. The lipids were dissolved in 1 mL extrusion buffer (5 mM Tris-HCl, pH 7.5, 40 mM NaCl, 10 mM MgCl_2_) and the suspension was vortexed for 5 min, followed by sonication for 10 min. The suspension was then extruded using a Mini Extruder Kit (Avanti Polar Lipids). The suspension was passed through a 100 nm membrane 41 times to produce a clear suspension of SUVs.

### 2.5. Evaluation of Membrane Binding Using Gel Shift Assay

Membrane binding was evaluated using a previously described gel-shift assay [42,43]. A measure of 10 µL of 1 nM purified DOT or DOB was incubated with 5 µL of SUVs (diluted 50× in extrusion buffer) at room temperature for 10 min. A measure of 10 µL of the mixture was analysed by electrophoresis (2% agarose gel in 0.5× TBE, supplemented with 11 mM MgCl_2_ and SyBr Safe DNA gel stain). All gels were run at 60 V for 2.5 h at RT. The gels were imaged using the Biorad Chemidoc MP Imager. All gel images were analysed using the Bio-Rad Image Lab software.

DOTs or DOBs that are not bound to the SUV migrate through the gel, while bound DOTs or DOBs remain in the wells. For each DNA nanostructure, the extent of membrane binding was evaluated by comparing the intensity of unbound DOT or DOB band when incubated with SUVs to the control DOT or DOB band when not incubated with SUVs. The extent of membrane binding is given by:

% bound = (1 − B/U) × 100%(1)

where % bound is the estimated percentage of membrane bound DOT or DOB, U is the intensity of the DOT or DOB band in the lane without SUVs, and B is the intensity of the DOT or DOB band in the lane with SUV. Two gel repeats were performed. Further details on the gel-shift assay are given in Figure S8. 

### 2.6. Transmission Electron Microscopy

For imaging of the bare DOTs and DOBs, 15 µL droplets of purified DOT or DOB solution (1 nM in folding buffer) was placed onto parafilm. A plasma-treated carbon-coated TEM grid (Ted Pella EM grids from ProScitech, Kirwan, QLD, Australia) was placed on the sample and left for 1 min. The sample was blotted using filter paper. Staining was then conducted by tapping the grid onto a droplet of 2% uranyl acetate solution on a parafilm for approximately 1 second and blotting. This staining protocol was repeated three times.

For imaging of the DOTs and DOBs bound to SUVs, 10 µL of 2 nM DOT or DOB solution was mixed with 5 µL of SUV solution (diluted 50× in extrusion buffer) and left at RT for 5 mins. A measure of 10 µL of the sample was then dropped onto a parafilm. A plasma-treated carbon-coated TEM grid (Ted Pella EM grids from ProScitech) was dropped onto the sample and left for 10 min. The sample was blotted using filter paper. The grids were then washed by tapping onto a droplet of Milli-Q and blotting. This washing step was repeated three times for each grid. Staining was then conducted by first tapping the grid onto a droplet of 2% uranyl acetate solution on a parafilm for approximately 1 second and blotting, and then dipping the grid into 2% uranyl acetate solution for approximately 10 seconds and blotting. The grids were left to air dry for at least 1 h prior to imaging. All TEM imaging was performed using the JEOL JEM-1400 microscope, 120 kV using a spot size of 2 and magnification between 50,000 and 100,000.

## 3. Results and Discussion

In this work, we tested the aggregation of two distinct DNA origami nanostructures: DNA origami tile (DOT) [1] and DNA origami barrel (DOB) [36]. We selected the DOT and DOB for this study as these nanostructures fold with high yield and are modular, as they have regularly spaced staple patterns, allowing them to serve as 2D and 3D molecular pegboards. Their modularity and reliability make them ideal for cholesterol labelling. Furthermore, their structural differences, that is the DOT being flexible [44] with a large surface area while the DOB being rigid and compact, allows us to test the effect of shape and rigidity on aggregation and membrane binding.

### 3.1. Effect of Number and Separation of Cholesterols, Spacer Length and Overhang on Aggregation of DOT

**Figure 2:**
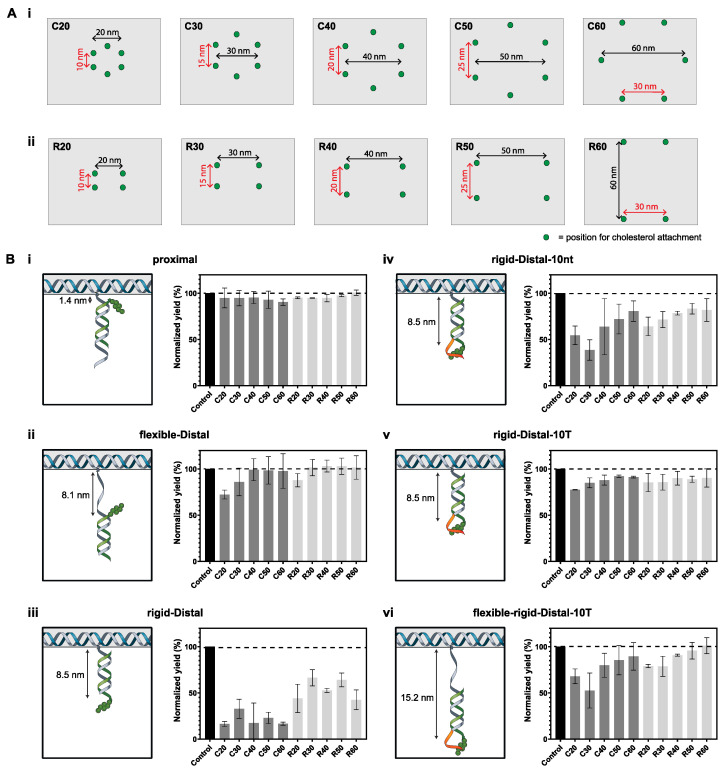
Systematic study of the aggregation of the DOT. (**A**). Top view of the DOT showing the position of cholesterol attachment for the different configurations tested. (**i**) Circular configuration. (**ii**) Rectangular configuration. (**B**). Schematic of the different spacer designs tested and their normalized yield of folding for the different configurations. A lower yield signifies more aggregation. (**i**) Proximal. (**ii**) Flexible-distal. (**iii**) Rigid-distal. (**iv**) Rigid-distal-10nt. (**v**) Rigid-distal-10T. (**vi**) Flexible-rigid-distal-10T. Staple extension from DOT: grey. Cholesterol strand: green. Overhang: orange.

First, we tested the effect of number and separation of the cholesterols on aggregation of the DOT by designing two different configurations of cholesterol placement: rectangular and circular (Figure 2A). The DOT was designed to be decorated by four and six cholesterols, in the rectangular and circular configurations, respectively. Four to six cholesterols were selected as we have previously shown that the membrane binding of the DOT is maximum when labelled with four to eight cholesterols [37]. Due to the staple nick pattern of the DOT, six instead of eight cholesterols were selected in the circular configuration to allow for even separation between the cholesterol groups.

We varied the separation between the cholesterols to study the effect on the aggregation of the DOT. For the circular configuration, the diameter of the cholesterol arrangement was varied between 20 nm and 60 nm (Table 1). Similarly, in the rectangular configuration, the separation of the cholesterols on the longer edge was varied between 20 nm and 60 nm (Table 2). An overview of the position of cholesterol attachment across all the different configurations tested is given in Figure 2A.

We also tested the effect of the spacer length between the cholesterol and the DOT. A previous study showed that distally placed cholesterols are more likely to induce aggregation compared to proximally placed cholesterols [14]. In this work, we extend this knowledge by testing different spacer lengths and also testing different types of spacers (flexible ssDNA vs rigid dsDNA) and different overhangs (no overhang vs. 10-nt overhang vs. 10-T overhang). Furthermore, we test this for all the different configurations listed in Table 1 and Table 2. 

In total, we tested six different spacers*: proximal, flexible-distal, rigid-distal, rigit-distal-10nt, rigid-distal-10T and flexible-rigid-distal-10T* (Table 3 and Figure 2B). In the *proximal* design, there was no spacer and the only spacing between the cholesterols and the DOT was the TEG linker used to link the cholesterol to DNA. Here, the maximum spacing between the cholesterol and the DOT is estimated to be 1.4 nm [42]. DNA maximum spacer lengths are estimated using values of 0.34 nm/bp for dsDNA [45] and 0.67 nm/nt for ssDNA [46]. In the *flexible-distal* design, a 10nt ssDNA spacer was used, resulting in a maximum spacing of 8.1 nm. In the *rigid distal*, *rigid-distal-10nt* and *rigid-distal-10T* designs, a 21 bp dsDNA spacing was used, resulting in a maximum spacing of 8.5 nm. The *rigid-distal*, *rigid-distal-10nt* and *rigid-distal-10T* designs have no overhang, a 10nt overhang and a 10-T overhang, respectively. Finally, in the *flexible-rigid-distal-10T* design, the 10-nt flexible ssDNA and the 21bp rigid dsDNA spacings were combined to result in a larger spacing between the cholesterols and the DOT, with the maximum spacing being 15.2 nm. The *flexible-rigid-distal-10T* design also has a 10-T overhang.

For all configurations and spacer designs, the DOTs were folded in one-pot and aggregation was analysed by gel electrophoresis (Figures S1 and S2). Two repeats of DOT folding and gel analysis was conducted. Gel electrophoresis provides a high throughput method to determine the yield of the DOT, and typically has a scan-to-scan variability of <10% RSD [47] (relative standard deviation). The absolute yield of the DOT band as a percentage of the scaffold DNA added was determined by evaluating the DOT band intensity as a percentage of the total lane intensity above the band. The absolute yield of the control band was observed to vary between gels. Thus, to compare between gels, a normalized yield was calculated for each design by normalizing to the absolute yield in the control (no cholesterol) lane. A higher normalized yield indicates lower aggregation. All yields as tabulated from gel images are given in Tables S1–S3. All yields below refer to normalized yields unless otherwise stated.

For the *proximal* spacer design, a high yield was observed for all configurations, with yields in the range of 90–95% for the circular configurations and 95–101% for the rectangular configurations (Figure 2B(i)), suggesting minimal aggregation. This was expected based on previous studies, which show low aggregation for proximal placement [14] but also lower membrane-binding yield [37]. Note that a yield of over 100% is possible as these yields are normalized to the control lane, and gel loading and analysis error can be up to 10% RSD [47].

In contrast, for the distal designs, the yield was found to depend both on cholesterol spacing (distance between tile and cholesterol) and separation (distance between adjacent cholesterols on tile). For the *flexible-distal* (8.1 nm spacer) design, the lowest yield of 72 ± 5% (i.e., greatest aggregation) was observed for the C20 configurations, which has adjacent cholesterol separation of 10 nm, followed by a yield of 86 ± 15% for the C30 configuration which has an adjacent cholesterol separation of 15 nm (Figure 2B(ii)). The yield range for the other circular configurations (C40–C60) was 98–99%. For the rectangular configurations, the lowest yield was for R20 at 88 ± 7%, with the yield of the other configurations in the range of 101–103% (R30–R60). 

For the *rigid-distal* (8.5 nm spacer) design, yields were lower than for *flexible-distal* or *proximal* designs. The lowest yield (16 ± 3%) was observed for the C20 configurations, which has adjacent cholesterol spacing of 10 nm (Figure 2B(iii)). Overall, for this design, lower yields of 17–33% were observed for the other circular configurations (C30–C60) and 43–66% for the rectangular configurations (R20–R60) (Figure 2B(iii)). The lowest yield for the rectangular configuration was observed for R60 (43 ± 11%). No clear trend in the effect of cholesterol spacing was observed here. However, the difference between the circular and rectangular configurations is significant (*p* < 0.05, unpaired *t*-test).

**Figure 3:**
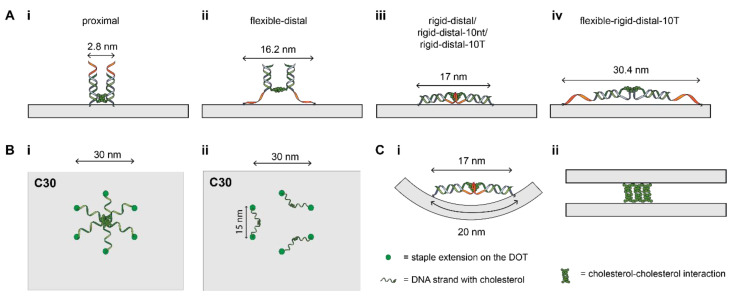
Schematic showing potential interactions between cholesterols (green) that could promote aggregation of the DOT (grey). (**A**). Schematic showing the maximum distance between cholesterols at which they could interact for the different spacer designs. This assumes the DOT is flat, if the DOT is curved or folded then the distances would be smaller (**Ci**); (**B**). Possible intra-DOT cholesterol interactions. (**i**) Interaction between all six cholesterols in the circular configuration. (**ii**) Interactions between adjacent cholesterols; (**C**). Possible intra-DOT and inter-DOT interactions. (**i**) The flexible DOT structure could bend to allow for cholesterols on its surface to interact even when the planar distance between the cholesterols on the surface of DOT exceeds the distance required for them to interact. (**ii**) Cholesterols on different DOTs can interact to form higher order structures.

It has been previously shown that distally placed cholesterols result in greater aggregation [28]. Our results agree with this as the yields for *flexible-distal* and *rigid-distal* designs were lower than for either the *proximal* spacer design or the no cholesterol control. Interestingly, for the two distal spacer designs, there was a clear difference in yield for the different configurations. We consider the relationship between the predicted separation between adjacent cholesterol groups based on their position on the DOT and predicted spacer length, and yield of that design. For the *flexible-distal* design the total spacer length expected for two adjacent cholesterols is 16.2 nm (Figure 3A(ii)). For designs with cholesterol separation < 15 nm (C20, C30, R20), the largest yield decrease was observed, while for separation > 20 nm (C40–C60, R30–R60), the yield decreased by a smaller amount. 

Similarly, for *rigid-distal* the total linker spacing is approximately 17 nm (Figure 3A(iii)). However, no clear trend was observed for either the circular or rectangular configurations, with the lowest yield for the circular configuration observed when the separation between adjacent cholesterols was 10 nm (C20) (Figure 2A(i)) and the lowest yield for the rectangular configuration for R60 (Figure 2A(ii)). Our results show that the *rigid-distal* design is generally very prone to aggregation, with all configurations having a low yield, and this effect dominates over differences between configurations. Therefore, our results suggest that interactions between adjacent cholesterols may be a mechanism for aggregation for designs where the cholesterol separation is a close match to the total spacer length. However, as the DOT structure is flexible and can bend, the actual distances bridged by cholesterols may be smaller than expected (Figure 3C(i)), and this parameter does not account for all yield variations we observed. 

We found that adding an overhang proximal to the cholesterol group increased the yield of the DOT assembly. For the *rigid-distal-10nt* design, where a 10-nt overhang containing a mix of DNA nucleotides was used, the average yield for the circular configuration was 62 ± 20%, compared to 21 ± 11% with no overhang. The average yield for rectangular configurations was 76 ± 10% (Figure 2B(iv)), compared to 54 ± 13% with no overhang. For the *rigid-distal-10T* design, where a 10-T overhang was used, the average yield for the circular configurations was 87 ± 6% while the average yields for rectangular configurations was 88 ± 6% (Figure 2B(v)). This suggests that for minimizing aggregation of DNA origami-sized nanostructures (>7000 bp), an overhang is better than no overhang, and a 10-T overhang is more effective as compared to a 10-nt overhang of random sequence. Analysis of the overhangs by Nupack [48] showed that neither of the sequences is predicted to form a secondary structure (Figure S4). These results are consistent with previous studies on much smaller systems, such as individual DNA strands (<100 bp) and 4-helix bundles [29] (<500 bp) that observed a sequence-dependent minimization of aggregation, with 10-T giving the greatest reduction in aggregation. Interestingly, when a 10-nt overhang was used, some separation dependence was again observed, with the lowest yields observed for C30 (39 ± 11%). 

In contrast, for the *flexible-rigid-distal-10T* (15.2 nm spacer) design, the 10-T overhang was not able to completely minimize aggregation. We found that the lowest yield was observed for the C30 configuration (53 ± 19%, Figure 2B(vi)) compared to 85 ± 5% for *rigid-distal-10T*. This may be due to the longer spacer length (15.2 nm) as compared to the *rigid-distal-10T* design (8.5 nm). As such, the total spacer length expected for two adjacent cholesterols is 30.4 nm (Figure 3A(iv)). This potentially corresponds to cholesterol groups aggregating in the center of the circular configuration (Figure 3B(i)) in the C30 configuration. Therefore, while a poly-T overhang can reduce aggregation, it is not the ultimate solution for every cholesterol-DNA nanostructure design, particularly those with longer spacer designs that incorporate flexible ssDNA regions, such as toehold regions for DNA strand displacement [37]. 

For all spacer designs, the mean yield for the rectangular configuration was higher than the circular configuration (Figure S3). This was statistically significant (unpaired *t*-test, *p* < 0.005) for the *rigid-distal* design, where the average yield for all the circular configurations was 21 ± 11%, while the average yield for the rectangular configuration was 54 ± 13%, which is a 2.5-fold increase compared to the circular configuration. This suggests that reducing the number of cholesterols can significantly reduce the aggregation of the DNA origami-sized nanostructures, in agreement with results for smaller DNA 4 helix-bundle structures [29].

### 3.2. Effect of Spacer Length and Overhang on Aggregation of DOB

**Figure 4:**
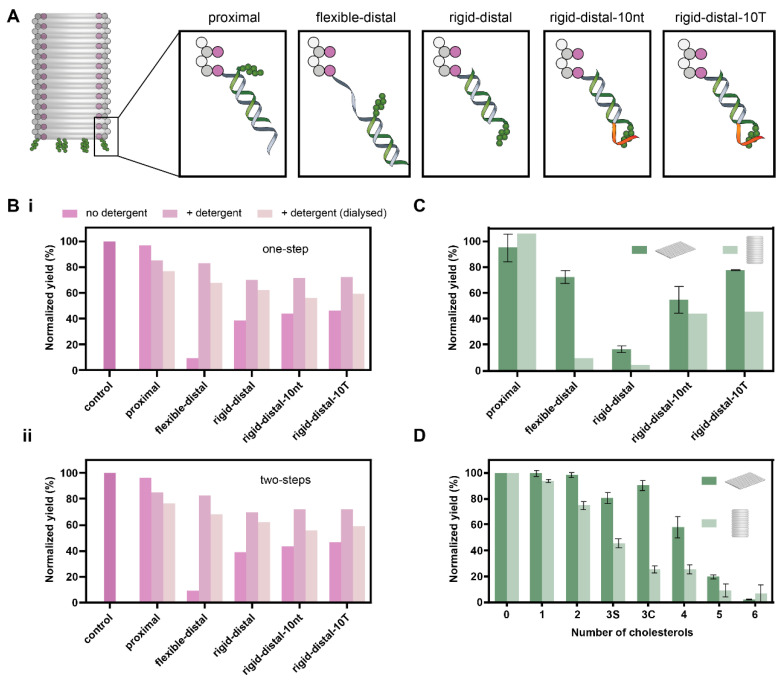
Aggregation study of the DOB. (**A**). Schematic showing the different spacer designs for cholesterol attachment. In all cases, the staple (grey) from the DOB is extended from its inner helix. Cholesterol strand: green. Overhang: orange; (**B**). Normalized DOB yields based on gel analysis for (**i**) one-pot and (**ii**) two-step cholesterol attachment. A lower yield indicates more aggregation; (**C**). Comparison of normalized yield of DOT and DOB. Gel bands for each design were normalized to the control band in the gel. One-pot folding results (no detergent) were used for the DOB; (**D**). Normalized yields of the DOT and DOB with different number of cholesterols.

Next, we tested the effect of the spacer length and overhang type on the aggregation of a 3D nanostructure, the DOB. Five different designs were compared: *proximal, flexible-distal, rigid-distal*, *rigid-distal-10nt* and *rigid-distal-10T* (Figure 4A). The handles for cholesterol attachment were extended from the inner layer of the DOB wall. 

Three-dimensional DNA nanostructures are known to be more prone to aggregation and misfolding [36,40]. Therefore, we expected greater aggregation with the cholesterol-modified DOB. Previous studies using cholesterol-labelled 3D DNA nanostructures have used the addition of cholesterols in a second folding step [7,16] and the use of detergent such as OG [49,50] to minimise aggregation. Therefore, we tested one- and two-step folding, and compared three different detergent conditions for each: no detergent, 1% OG, and 1% OG (dialysed post folding). In one-pot folding, all strands were added and folded either with or without 1% OG. For the dialysed sample, the detergent was removed post-folding by overnight dialysis. In the two-step folding, the DOB was folded with all strands except for the cholesterol-modified strands. Post-folding, cholesterol-modified strands (pre-heated to 37 °C) were added with or without 1% OG and incubated for 1 h at 37 °C. Again, for the dialysed sample, the OG detergent was removed by overnight dialysis.

We found that for both one-pot and two-step folding, the addition of detergent improved the yield of the DOB. However, in all cases, the yield of the DOB decreased again upon removal of detergent by dialysis. A normalized yield of 106% was observed for the *proximal* design, and yields of 4–46% were observed for the distal designs, respectively (Figure 4B(i) and Figure S5, Table S3), for one-pot folding with no detergent. The use of detergent resulted in an increase in normalized yield for the distal designs (78–106%). However, these yields decreased (72–78%) upon dialysis. Therefore, similarly to the DOT, we found that the *proximal* design was most optimal for one-pot folding of the DOB.

The two-step protocol resulted in slightly lower yields. Without detergent, a normalized yield of 97% was observed for the *proximal* design and between 9–46% for the distal designs (Figure 4B(ii) and Figure S5, Table S3). The addition of detergent resulted in increased normalized yields for all distal designs (70–83%). However, these yields decreased (56–68%) upon dialysis.

The results suggest that a two-step protocol does not reduce the cholesterol-induced aggregation of this 3D DNA nanostructure. In all cases, the *proximal* design resulted in the highest yields. While the addition of detergent improved the yield of all the distal designs, their yields were still lower than the *proximal* design. The improved yield also decreased upon dialysis, a step which is necessary if these nanostructures are to be used with lipid membranes as detergent can solubilize lipid membranes [51]. Overall, for these results, the spacer length was the most important parameter in minimizing the aggregation of the 3D DNA nanostructure DOB.

### 3.3. Comparison of Yields of DOT and DOB

We next compared the yields of the DOB against the DOT with C20 configuration (Figure 4C). The C20 configuration was selected for comparison as the diameter of the handle extension in this configuration is comparable to the DOB. For each design, we calculated the normalized yield of the DOT or DOB band as normalized by the no-cholesterol control band. For the *proximal* design, a higher yield was observed for the DOB at 106% compared to 95 ± 11% for the DOT. For all other designs, the yield of the DOT was higher than the yield of the DOB.

Next, we compared the effect of number of cholesterols on the aggregation of the DOT and the DOB. We selected the *rigid-distal* design for this comparison as it previously resulted in the lowest yield (Figure 2B). We varied the number of cholesterols from zero to six, and compared two different arrangements for three cholesterols: clumped (3C) and sparse (3S). The arrangement of cholesterols is given in Figure S6. We found that the yield decreased from zero to six cholesterols for both the DOT and the DOB (Figure 4D and Figure S7).

For the DOT, the change in yield was not significant for 0, 1, 2, 3C cholesterols. A significant decrease in yield (unpaired *t*-test, *p* < 0.005) was observed from three cholesterols (80 ± 4% for 3S, 91 ± 4% for 3C) all the way to six cholesterols (2 ± 0.2%). The difference between the 3C and 3S cholesterols was insignificant.

For the DOB, the difference between zero (100%) and one (94 ± 1) cholesterol was insignificant. Adding more than one cholesterol group resulted in a significant (unpaired *t*-test, *p* < 0.005) decrease in yield. A significantly lower yield (unpaired *t*-test, *p* < 0.005) was observed for 3C (25 ± 3%) compared to 3S (46 ± 3%). The yield difference between three cholesterols (3C) and four cholesterols was insignificant, as was the difference between five and six cholesterols.

### 3.4. Comparison of Membrane Binding of DOT and DOB

**Figure 5:**
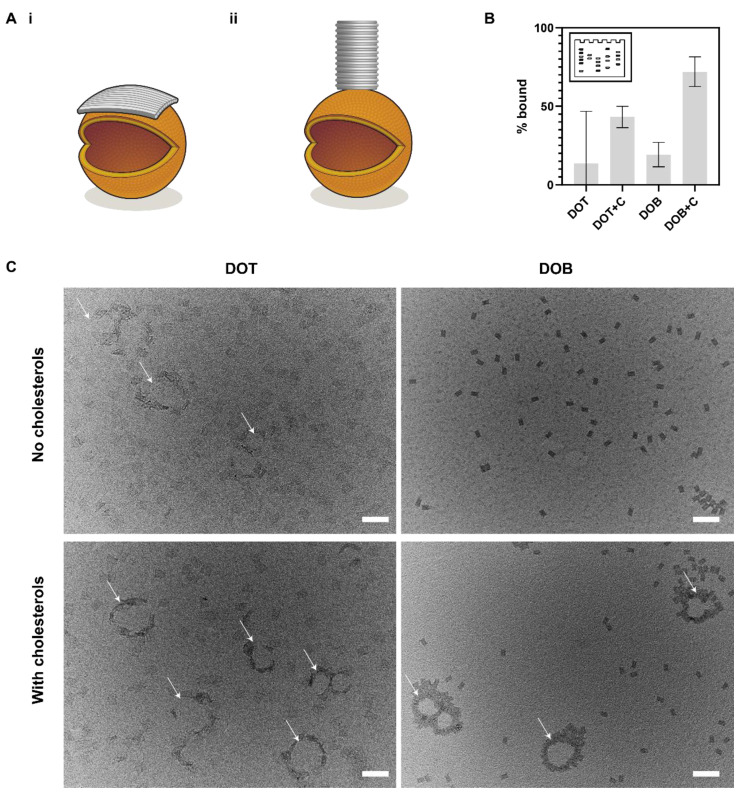
Membrane binding. (**A**). Schematic showing the binding of a DOT (**i**) and DOB (**ii**) to an SUV (orange) for the proximal spacer design; (**B**). Bar chart showing the % bound based on the gel-shift assay. The error bars are from two gel repeats (mean plus standard deviation); (**C**). TEM images showing the membrane binding of the DOT and DOB in the absence and presence of cholesterols. While liposomes are not visible in these conditions, clustering of DOT and DOB around circular voids indicate regions of liposome binding (white arrows). Scale bar: 200 nm.

Finally, we compared the membrane binding of the DOT and DOB to SUVs (Figure 5A). It has been previously shown that increasing the spacer length results in increased membrane binding [32,37]. However, in this work, we found that aggregation is minimized with the shortest possible spacer (*proximal* design). Therefore, membrane binding experiments were conducted with the *proximal* design for both the DOT and DOB. The C20 configuration was selected for the DOT as it is comparable to the cholesterol configuration on the DOB. Membrane binding was evaluated quantitatively using a previously established gel-shift assay [42,43] (Figure 5B and Figure S8) as well as qualitatively using TEM (Figure 5C). For both the DOT and DOB, membrane binding was compared with and without cholesterols.

For the cholesterol-modified samples, DOT+C and DOB+C, increased membrane binding was observed by TEM imaging compared with controls (Figure 5C). By gel, higher membrane binding was similarly observed for cholesterol samples as compared to the no-cholesterol controls (Figure 5B). For the unmodified DOTs, the percentage of bound DOTs was 14 ± 33%, increasing to 43 ± 7% for DOT+C. The percentage of membrane-bound DOBs and DOB+C were 19 ± 8% and 72 ± 10%, respectively. The percentage bound observed by gel for unmodified DOB and DOT was similar, but there was greater error in the DOT sample and some evidence of binding between the DOT and liposomes in TEM data (Figure 5C). This suggests the 2D DOT may be more likely to interact non-specifically with liposomes than the 3D DOB. Membrane binding of unmodified dsDNA to liposomes has been observed in the presence of divalent cations such as magnesium and calcium [52], and membrane binding of unmodified DOT has been shown previously in the presence of magnesium ions [14]. Our results suggest that nonspecific interactions between unmodified DNA origami nanostructures and membranes may be dependent on the size and shape of the nanostructure. 

The percentage bound observed for DOB+C was ~1.65x more than that of the DOT+C. This suggests that the cholesterols on the DOB may be more available compared to the cholesterols on the DOT. We have previously shown that the configuration of cholesterols on the DOT can affect membrane binding [37]. Thus, we also tested the membrane binding of different configurations of the DOT (C20–C60) to ensure that the lack of binding observed was not due to differences in configuration. The cholesterol configuration on the DOT did not significantly affect the membrane binding of the structure and all configurations resulted in lesser membrane binding compared to the DOB (Figure S9).

The DOT is a flexible nanostructure [44] and has been shown to fold into itself when labelled with cholesterols [14], which may limit membrane binding. In contrast, the DOB is rigid and unlikely to fold into itself. Furthermore, the DOT is more likely to saturate the surface of the SUVs as it has a large predicted surface area of 5400 nm^2^. Meanwhile, the DOB is likely to only occupy 700 nm^2^ on the SUV via cholesterol interaction end-on. If the DOB lies sideways on the SUV, this area would increase to 1560 nm^2^, although this orientation is unlikely as there are no cholesterols on the lateral surface of the DOB. We predict the surface area of the SUVs (extruded using a 100 nm membrane) to be approximately 31000 nm^2^. Therefore, while it would require approximately 6 DOTs to saturate the surface of a single SUV, up to 44 DOBs could occupy a single SUV.

The increased compactness of a DNA nanostructure has been previously shown to increase its cellular uptake [53]. However, our work is the first to evaluate the effect of shape on the membrane binding of cholesterol-labelled DNA nanostructures. Our findings are consistent with a recent study where it was shown that the shape of ssDNA-labelled DNA nanostructures affects its hybridisation to complementary-ssDNA-labelled cells, with a rod-shaped nanostructure resulting in greater hybridisation compared to a rectangular nanostructure [34]. We extend this finding by showing that the shape affects the membrane binding of cholesterol-modified DNA nanostructures as well.

## 4. Conclusions

In this work, we tested different design parameters to minimize the cholesterol-mediated aggregation of DNA origami nanostructures. We found that the spacer length, presence of an overhang next to the cholesterol group, number of cholesterols as well as the separation between adjacent cholesterols all play an important role in the aggregation of the nanostructures. 

We found the spacer length to have the biggest effect on the yield of the DOT (Figure S2). More proximal cholesterol configurations result in lower aggregation. On average, across the 10 configurations tested (C20–R60), normalized yields increased from 38 ± 20% for the *rigid-distal* design to 95 ± 5% for the *proximal* designs. Our results also show that placing a 10-T overhang next to the cholesterol groups can minimize aggregation; however, this effect diminished with increasing spacer length. Aggregation can also be minimized by decreasing the number of cholesterols. For the *rigid-distal* design, reducing the number of cholesterols from six to one resulted in an increase in yield from 2 ± 0.2% to 100 ± 2%. We also found that for a particular spacer length on the DOT, the separation of cholesterols on the DOT can be optimized to minimize aggregation. 

The spacer length also affected the aggregation of the 3D DOB, with significant aggregation observed in all but the *proximal* spacer design. For distal cholesterol configurations, the use of detergent improved the yield (26 ± 22% to 92 ± 12%), but samples re-aggregated after detergent removal (74 ± 3%). We therefore conclude the use of detergent as a less effective method to minimize aggregation. Similarly, a two-step folding protocol did not reduce aggregation of this design, and introduced more complexity to the assembly process. Similar to the DOT, reducing the number of cholesterols on the DOB from six to one also resulted in an increase in yield, from 7 ± 7% to 94 ± 1%.

Differences in aggregation behavior of the 2D DOT and the 3D DOB were also shown here. In all but the *proximal* design, the DOB is more prone to aggregation compared to the DOT with a similar cholesterol configuration. Finally, we showed that these nanostructures bind to membranes to different extents, with 65% greater membrane binding observed for the DOB compared to the DOT. We attribute this to the flexibility of the DOT, which allows it to fold into itself, thereby possibly decreasing membrane binding by sequestering cholesterol groups. The DOT also has a larger surface area, which potentially limits the number of individual DOTs that can bind to an SUV. 

Ultimately, this work provides general guidelines to minimise aggregation when designing cholesterol-modified DNA nanostructures. Future work could include extending this analysis to rigid 2D structures as well as flexible 3D structures of different shapes to further characterise the full range of cholesterol-induced aggregation of DNA nanostructures and their subsequent membrane binding. We envision that these findings will allow for the design and formation of more complex membrane-interacting DNA nanostructures with potential applications in nanomedicine and biophysics research such as cargo transport between lipid domains [54] or biocomputing on membranes.

## Figures and Tables

**Table 1:** Handle separation for cholesterol attachment in the circular configuration. The maximum separation is across the diameter of the circular configuration while the minimum separation is between adjacent cholesterols.

Configuration	Maximum Separation (Diameter), nm	Minimum Separation, nm
C20	20	10
C30	30	15
C40	40	20
C50	50	25
C60	60	30

**Table 2:** Handle separation for cholesterol attachment in the rectangular configuration. The maximum separation is along the long edge of the rectangular configuration while the minimum separation is along the short edge.

Configuration	Maximum Separation, nm	Minimum Separation, nm
R20	20	10
R30	30	15
R40	40	20
R50	50	25
R60	60	30

**Table 3:** Different spacer designs tested. The theoretical maximum spacing between the cholesterol and DOT is estimated here using 0.34 nm/bp for dsDNA [45], 0.67 nm/nt for ssDNA [46], and cholesterol-TEG linker length of 1.4 nm [42]. The actual spacing is likely to be smaller, especially for the flexible spacers.

Spacer	Spacer Type	Maximum Spacing, nm	Overhang
proximal	-	1.4	-
flexible-distal	10 nt ssDNA	8.1	-
rigid-distal	21 bp dsDNA	8.5	-
rigid-distal-10nt	21 bp dsDNA	8.5	10-nt
rigid-distal-10T	21 bp dsDNA	8.5	10-T
flexible-rigid-distal-10T	10 nt ssDNA + 21 bp dsDNA	15.2	10-T

## Data Availability

The data presented in this study are available in Supplementary Information.

## Supplementary Materials

The following are available online at https://www.mdpi.com/article/10.3390/membranes11120950/s1, Figures S1–S10, Tables S1–S4.
