# Supplementary material for: Minimizing Cholesterol-Induced Aggregation of Membrane-Interacting DNA Origami Nanostructures
Source: Membranes (Basel). 2021 Nov 30;11(12):950. doi: 10.3390/membranes11120950 (PMC8707602; doi:10.3390/membranes11120950)
Supplement: Supplementary file 1 [file membranes-11-00950-s001.zip › membranes-1418241_SI.pdf]

Minimizing Cholesterol-Induced Aggregation of Membrane-Interacting DNA Origami Nanostructures

Jasleen Kaur Daljit Singh [1,2,3], Minh Tri Luu [1,2,3], Jonathan F Berengut [1,3], Ali Abbas [2,3], Matthew AB Baker [5,6] and Shelley FJ Wickham [1,3,4]*

[1] School of Chemistry, The University of Sydney, Sydney, New South Wales, 2006, Australia.

[2] School of Chemical and Biomolecular Engineering, The University of Sydney, Sydney, 2006, New South Wales, Australia.

[3] The University of Sydney Nano Institute, The University of Sydney, Sydney, New South Wales, 2006, Australia

[4] School of Physics, The University of Sydney, Sydney, New South Wales, 2006, Australia.

[5] School of Biotechnology and Biomolecular Sciences, University of New South Wales, Sydney, New South Wales, 2052, Australia.

[6] CSIRO Synthetic Biology Future Science Platform, Brisbane, Australia.

* Correspondence: To whom correspondence should be addressed: shelley.wickham@sydney.edu.au

Supplementary Data

Supplementary Table S1: List of reagents used

Reagent	Supplier
All DNA strands	Integrated DNA Technologies (IDT)
M13mp18 ssDNA (p7249)	P-107, Bayou Biolabs
Tris-HCl	T3253, Sigma
NaCl	AJA465, Ajax-Finechem
$MgCl_2$	AJA296, Ajax-Finechem
EDTA	E9884, Sigma
MilliQ water	Milli-Q, Millipore
DOPE 18:1	850725P, Avanti Polar Lipids
DOPC 18:1	850375P, Avanti Polar Lipids
PE-biotin	870282P, Avanti Polar Lipids
SyBrSafe stain	S33102, Thermo Fisher Scientific
PEG 8000	P5413, Sigma
Tris base	FSBBP152-5, Fisher Scientific
Boric acid	B7901, Sigma
Agarose	BIO-41025, Bioline

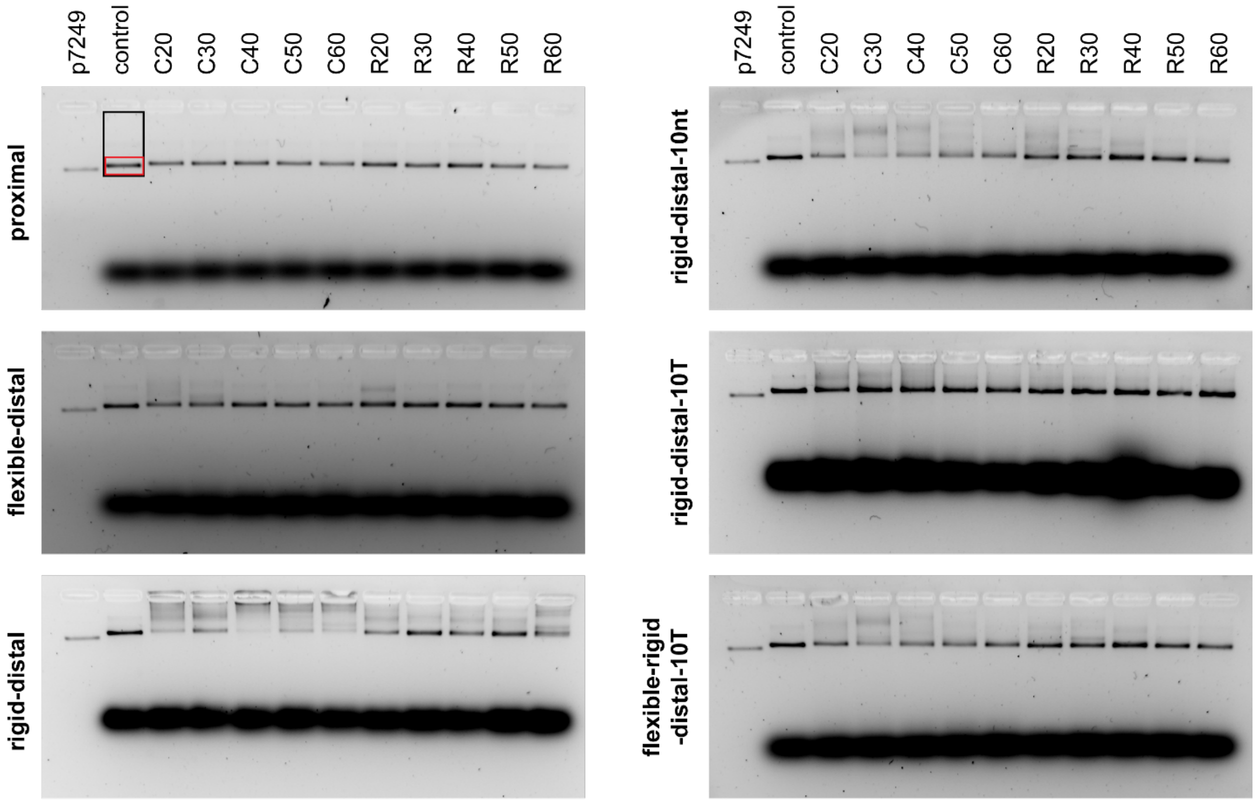

$$\text{absolute yield} = \frac{\text{intensity of band in red box}}{\text{intensity in black box}} \times 100\%$$

$$\text{normalized yield} = \frac{\text{absolute yield (lane of interest)}}{\text{absolute yield (control lane)}} \times 100\%$$

Supplementary Figure S1: DOT gels (repeat 1). Control lane is DOT folded with no cholesterols. For each lane, the yields of the DOT band is calculated as shown by the formula in the figure. Band intensities were determined using ImageLab software.

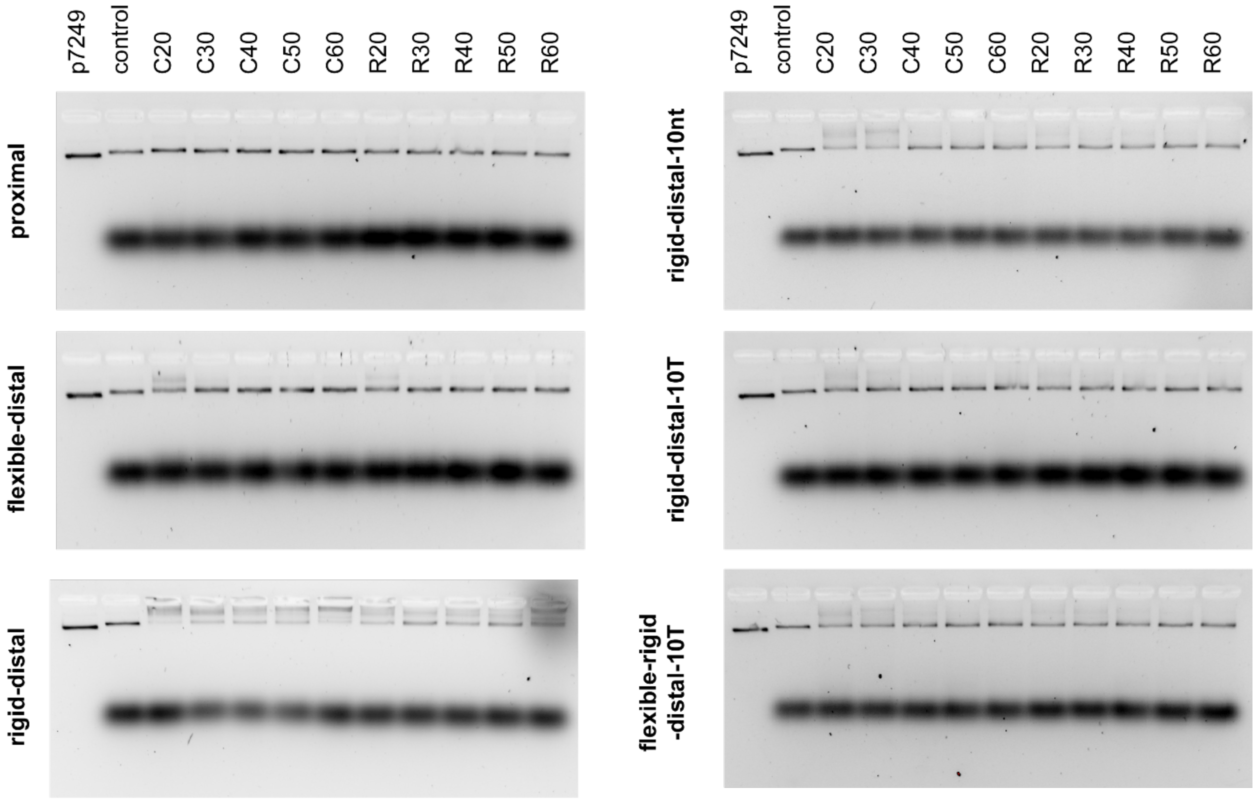

Supplementary Figure S2: DOT gels (repeat 2). Control lane is DOT folded with no cholesterols. For each lane, the yield of the DOT band is calculated as shown by the formula in the figure. Band intensities were determined using ImageLab software.

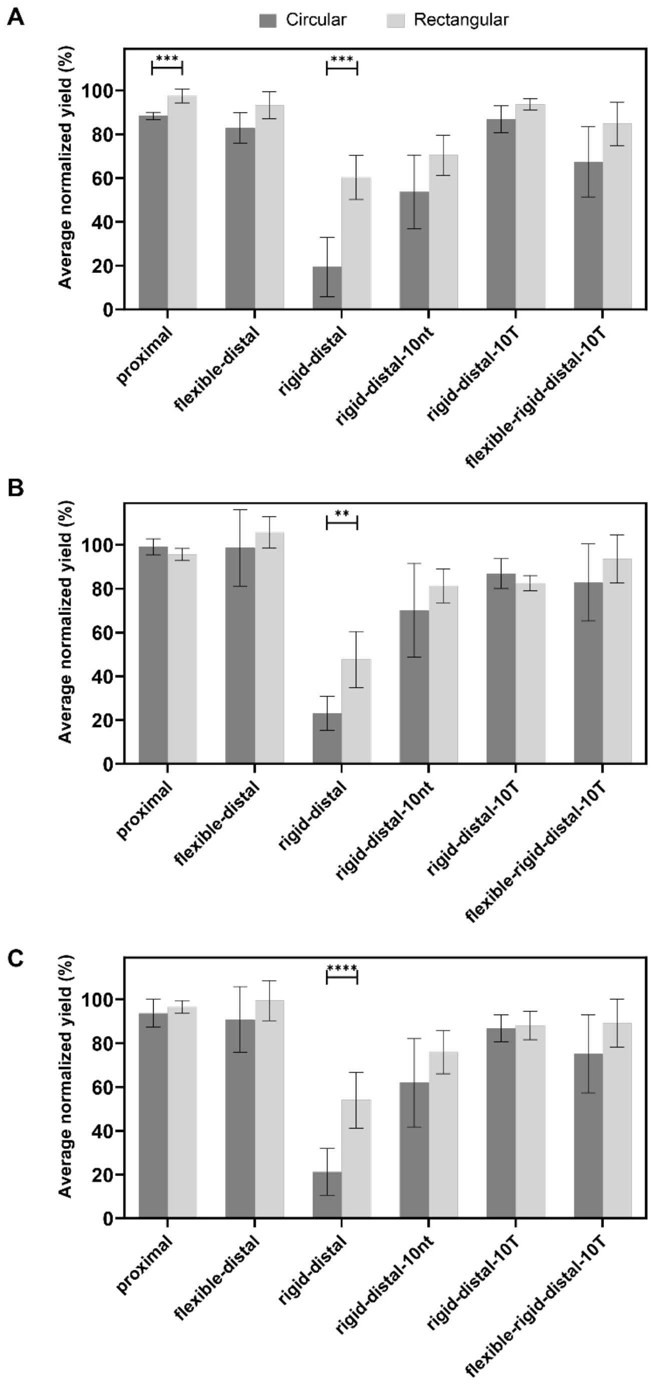

Supplementary Figure S3: Average yield for the rectangular and circular configurations on the DOT. The average is obtained by averaging the six different designs for each configuration. A. Average from gel repeat 1. B. Average from gel repeat 2. C. Combined averages from both gel repeats.

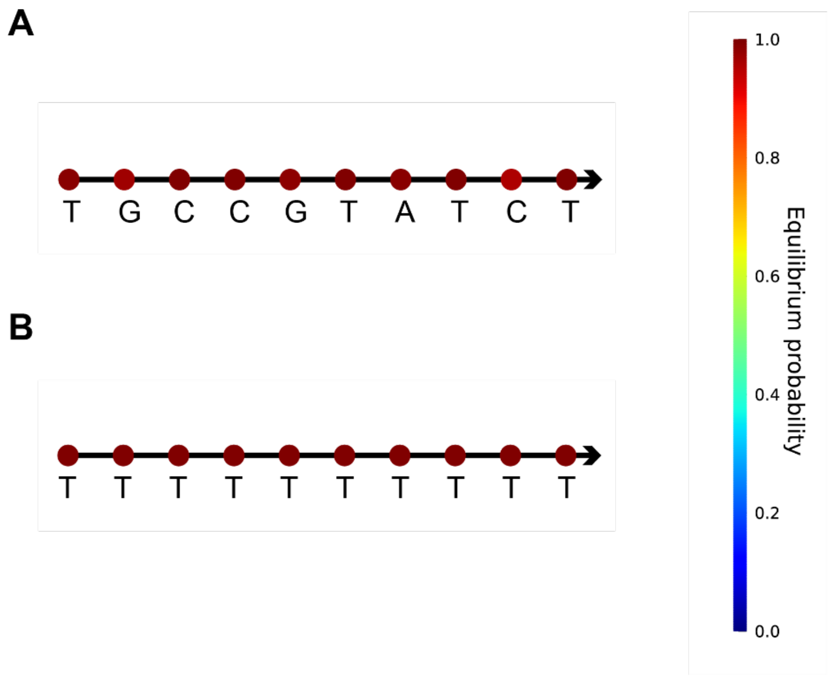

Supplementary Figure S4: Nupack analysis of the 10nt overhang (A) and 10T overhang (B). Maximum complex size was set to 2 to test for intra-strand binding, and concentration was set to 10 nm. The structural predictions shown are at 37°C. The stability of the base pairing is shown as a heat-map.

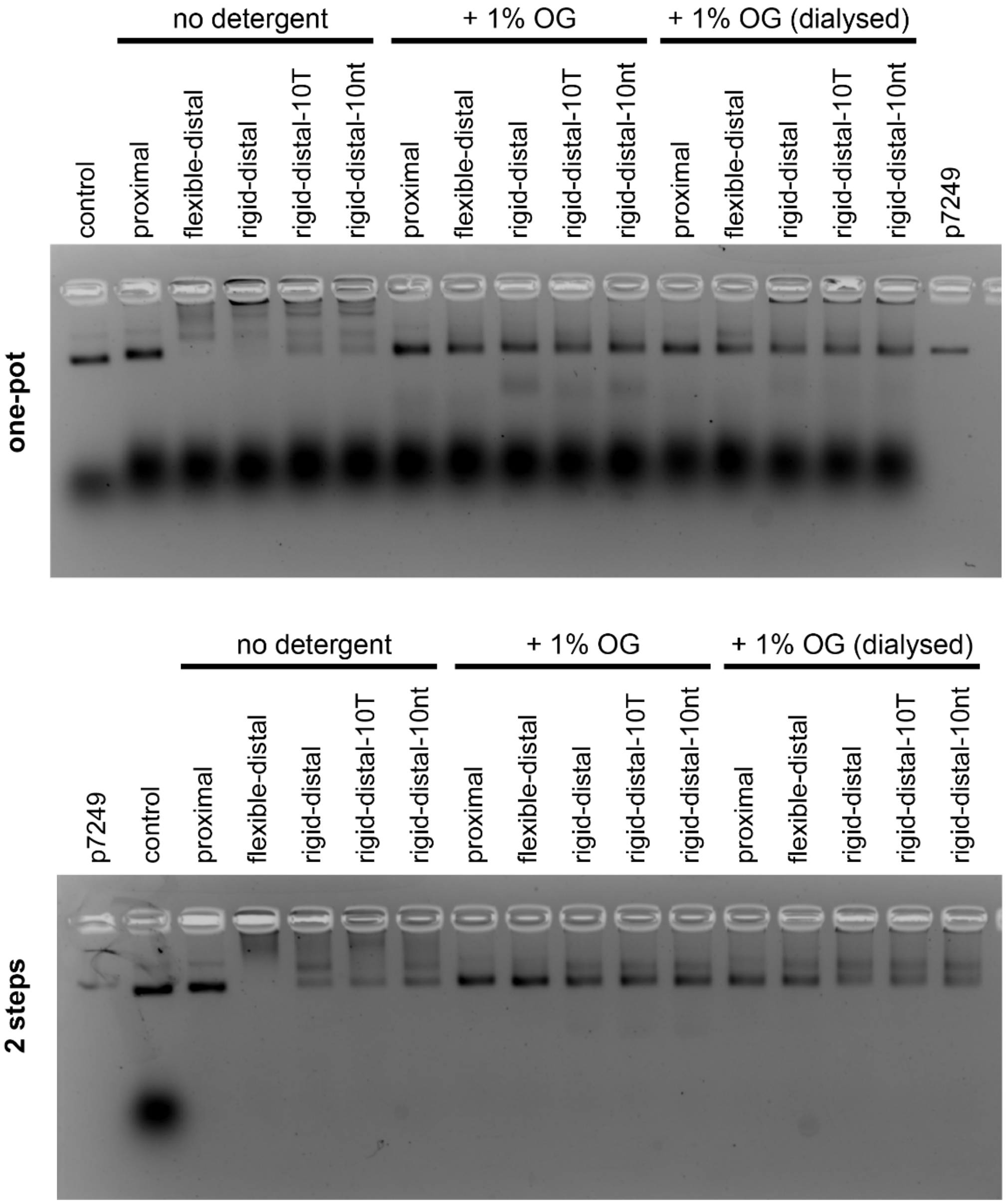

Supplementary Figure S5: DOB gels. Control lane is DOB folded with no cholesterols. For each lane, the yield of the DOB band is calculated, using the formula shown in Supplementary Figure 1.

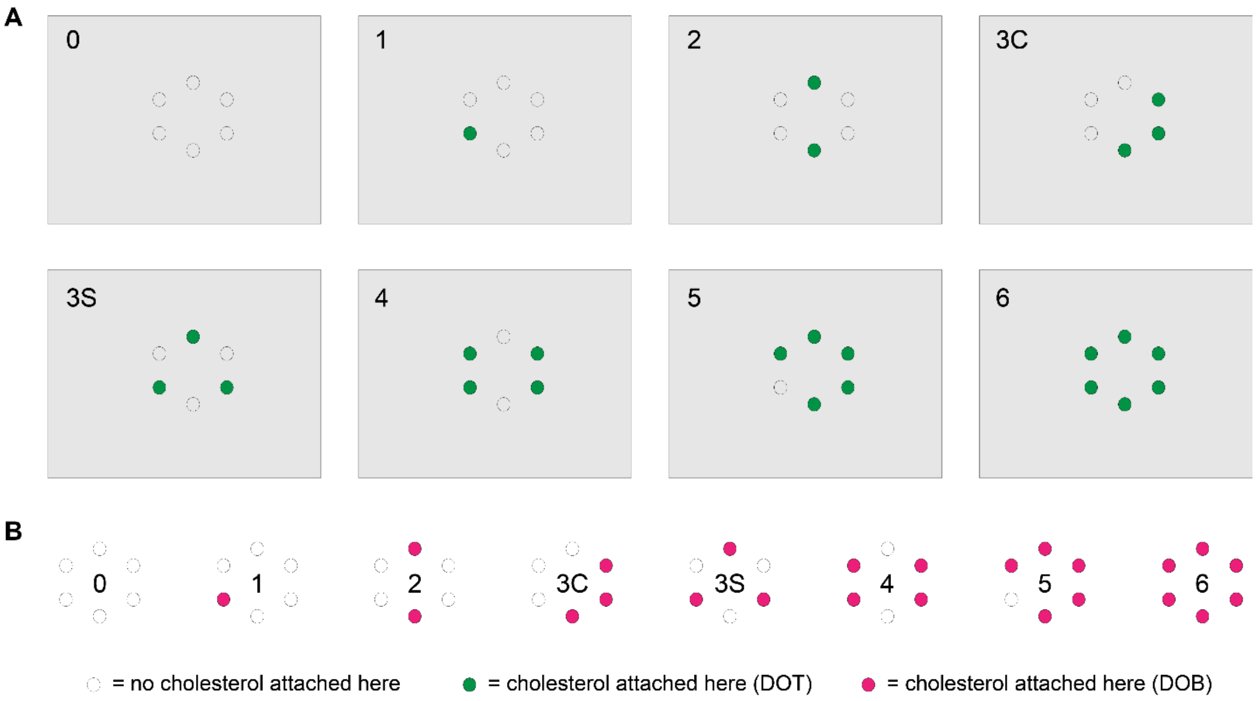

Supplementary Figure S6: Position of cholesterol placement on the DOT (A) and DOB (B). The number of cholesterols is stated in the figure. 3C = 3 cholesterols clumped, 3S = 3 cholesterols sparse.

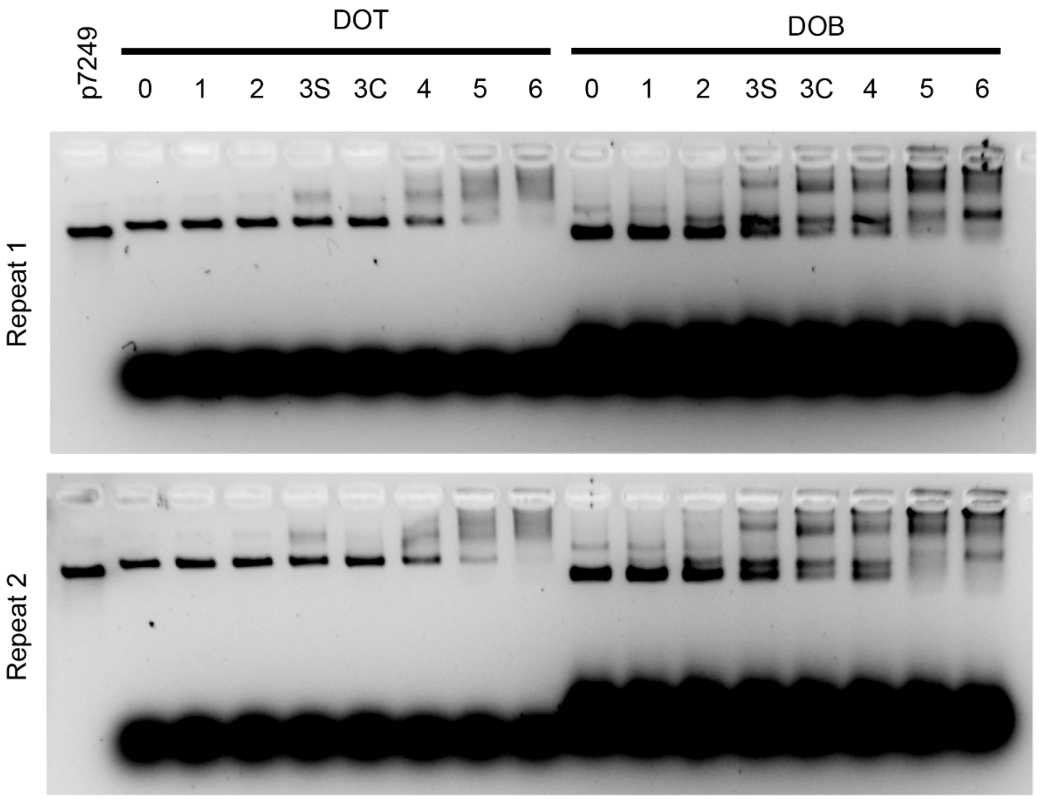

Supplementary Figure S7: Gel image DOT and DOB folded with different number of cholesterols. The number of cholesterols is annotated above the gel. 3C = 3 cholesterols clumped, 3S = 3 cholesterols sparse.

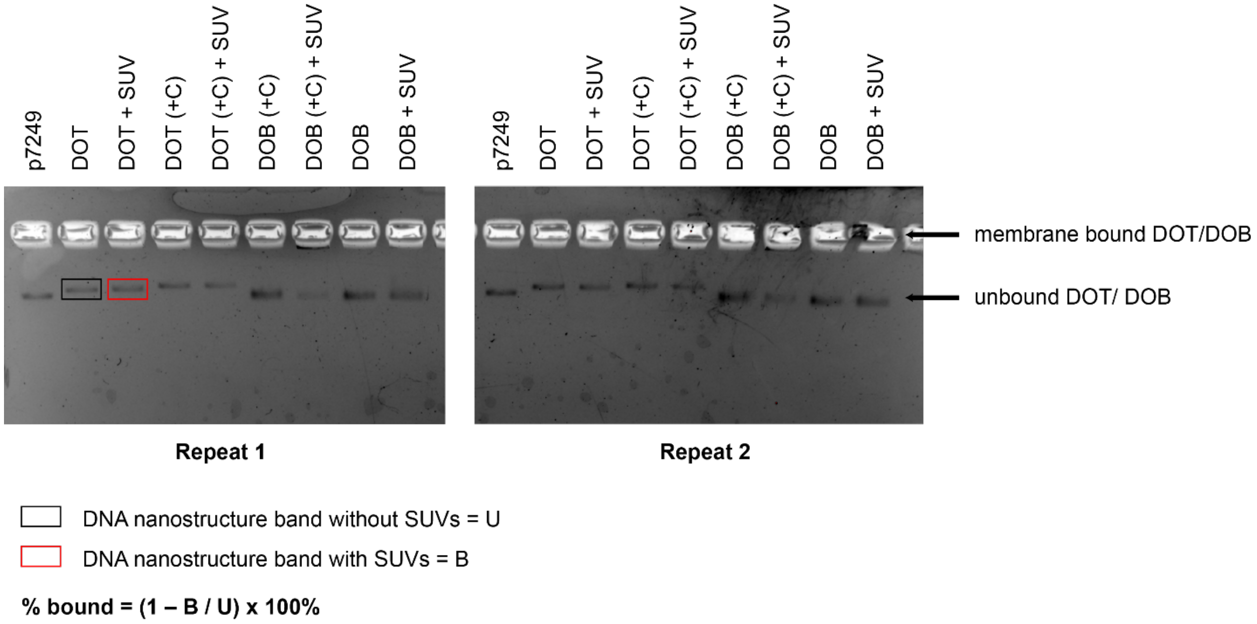

Supplementary Figure S8: Gel-shift assay to evaluate the membrane binding of DOT and DOB. The formula for calculating % bound is given.

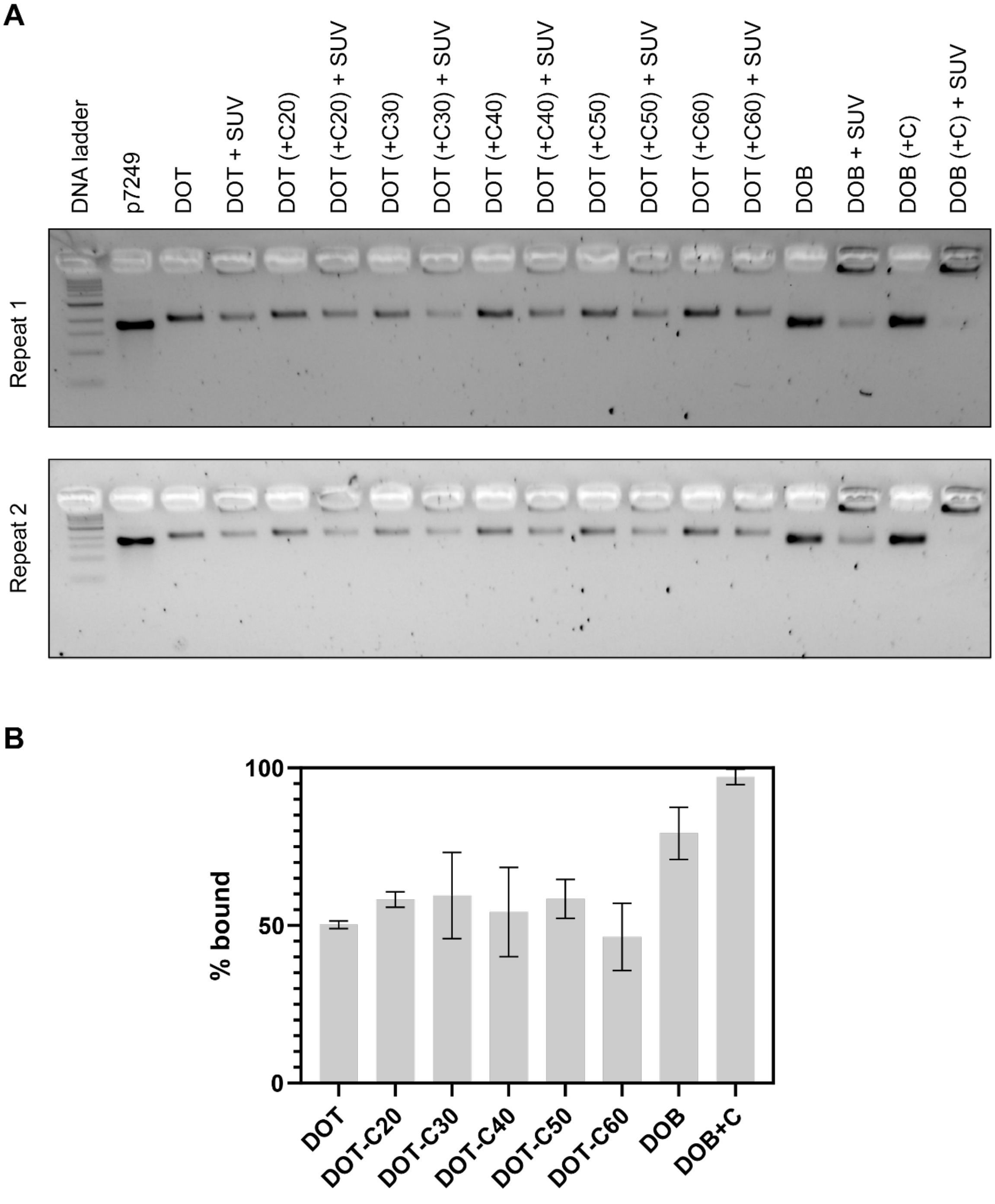

Supplementary Figure S9: A. Gel-shift assay to evaluate the membrane binding of different configurations of the DOT versus the DOB, for proximal spacer design. B. Percentage bound tabulated from the two gels.

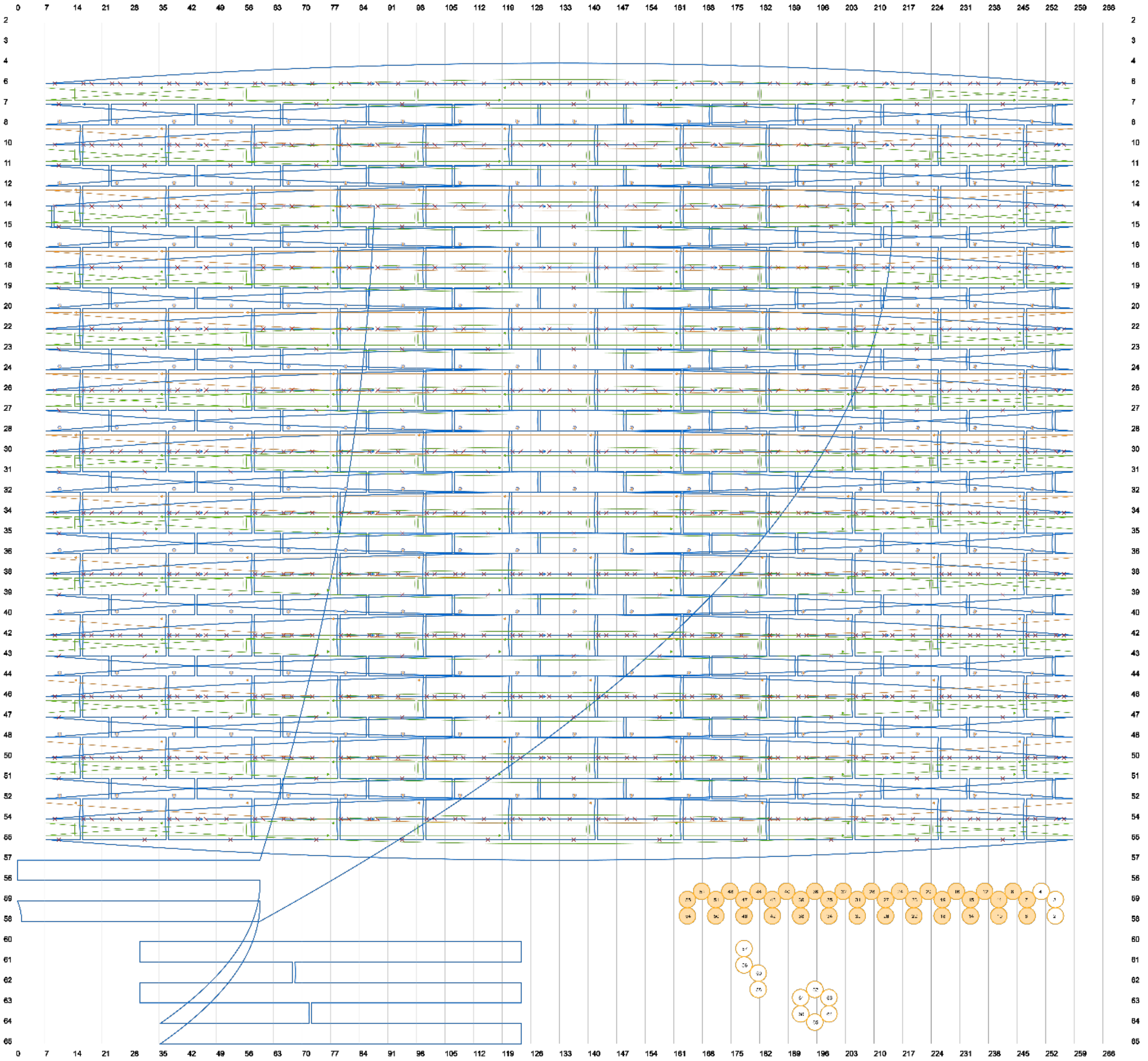

Supplementary Figure S10: Cadnano design for the DOB.

Supplementary Table S2: Absolute and normalized yields for the DOT samples. These are averages from the two repeats.

DOT *proximal*			DOT *rigid-distal*-10nt		
**Sample**	**Absolute yield (%)**	**Normalized yield (%)**	**Sample**	**Absolute yield (%)**	**Normalized yield (%)**
Control	80 ± 5	-	Control	85 ± 6	-
C20	76 ± 14	95 ± 11	C20	46 ± 5	54 ± 10
C30	76 ± 12	95 ± 8	C30	33 ± 12	39 ± 11
C40	76 ± 10	95 ± 6	C40	55 ± 30	64 ± 30
C50	74 ± 12	93 ± 9	C50	62 ± 18	72 ± 16
C60	72 ± 8	90 ± 3	C60	69 ± 15	81 ± 11
R20	76 ± 4	95 ± 1	R20	55 ± 13	64 ± 10
R30	76 ± 5	95 ± 0.3	R30	61 ± 12	72 ± 9
R40	75 ± 2	95 ± 4	R40	67 ± 7	79 ± 2
R50	78 ± 6	97 ± 1	R50	71 ± 10	83 ± 6
R60	80 ± 3	101 ± 3	R60	70 ± 16	82 ± 12
**DOT *flexible-distal***			**DOT *rigid-distal*-10T**		
**Sample**	**Absolute yield (%)**	**Normalized yield (%)**	**Sample**	**Absolute yield (%)**	**Normalized yield (%)**
Control	75 ± 6	-	Control	84 ± 2	-
C20	54 ± 1	72 ± 5	C20	65 ± 2	78 ± 0.3
C30	65 ± 16	86 ± 15	C30	72 ± 3	85 ± 5
C40	74 ± 15	99 ± 12	C40	74 ± 6	88 ± 5
C50	74 ± 17	99 ± 15	C50	78 ± 0	92 ± 1
C60	74 ± 20	98 ± 19	C60	77 ± 2	91 ± 1
R20	66 ± 11	88 ± 7	R20	72 ± 7	85 ± 10
R30	76 ± 13	101 ± 9	R30	72 ± 6	86 ± 9
R40	77 ± 11	103 ± 6	R40	76 ± 5	90 ± 7
R50	77 ± 13	103 ± 9	R50	75 ± 1	89 ± 3
R60	76 ± 16	102 ± 13	R60	76 ± 7	90 ± 10
**DOT *rigid-distal***			**DOT *flexible-rigid-distal*-10T**		
**Sample**	**Absolute yield (%)**	**Normalized yield (%)**	**Sample**	**Absolute yield (%)**	**Normalized yield (%)**
Control	82 ± 9	-	Control	78 ± 2	-
C20	13 ± 1	16 ± 3	C20	53 ± 8	68 ± 8
C30	26 ± 5	33 ± 10	C30	41 ± 13	53 ± 19
C40	15 ± 19	17 ± 22	C40	62 ± 8	80 ± 13
C50	19 ± 7	23 ± 6	C50	66 ± 10	85 ± 16
C60	14 ± 0.2	17 ± 2	C60	69 ± 9	90 ± 15
R20	35 ± 8	44 ± 15	R20	61 ± 0.5	79 ± 2
R30	54 ± 1	66 ± 9	R30	61 ± 6	79 ± 11
R40	43 ± 3	53 ± 2	R40	71 ± 1	91 ± 1
R50	52 ± 0.1	64 ± 7	R50	74 ± 5	96 ± 9
R60	34 ± 5	43 ± 11	R60	78 ± 4	101 ± 9

Supplementary Table S3: Absolute and normalized yields for all the DOB samples.

DOB 1-step			DOB 2-steps		
**Sample**	**Absolute yield (%)**	**Normalized yield (%)**	**Sample**	**Absolute yield (%)**	**Normalized yield (%)**
**No detergent**			**No detergent**		
Control	49	-	Control	45	-
Proximal	52	106	Proximal	44	97
flexible-distal	5	10	flexible-distal	4	9
rigid-distal	2	4	rigid-distal	18	39
rigid-distal-10T	22	44	rigid-distal-10T	20	44
rigid-distal-10nt	23	46	rigid-distal-10nt	21	46
**Detergent added**			**Detergent added**		
Proximal	55	112	Proximal	39	85
flexible-distal	43	87	flexible-distal	38	83
rigid-distal	52	106	rigid-distal	32	70
rigid-distal-10T	39	78	rigid-distal-10T	33	72
rigid-distal-10nt	48	97	rigid-distal-10nt	33	72
**Detergent added, then removed**			**Detergent added, then removed**		
Proximal	53	107	Proximal	35	76
flexible-distal	36	73	flexible-distal	31	68
rigid-distal	35	72	rigid-distal	28	62
rigid-distal-10T	36	73	rigid-distal-10T	26	56
rigid-distal-10nt	39	78	rigid-distal-10nt	27	59

Supplementary Table S4: Absolute and normalized yields for the DOT and DOB folded with different number of cholesterols. These are averages from the two repeats.

**DOT**			**DOB**		
**Number of cholesterols**	**Absolute yield (%)**	**Normalized yield (%)**	**Number of cholesterols**	**Absolute yield (%)**	**Normalized yield (%)**
0	81 ± 3	-	0	79 ± 1	-
1	80 ± 1	100 ± 2	1	74 ± 0	94 ± 1
2	79 ± 2	98 ± 2	2	59 ± 2	75 ± 3
3S	65 ± 1	80 ± 4	3S	36 ± 3	46 ± 3
3C	73 ± 0.1	91 ± 4	3C	20 ± 2	25 ± 3
4	47 ± 5	58 ± 8	4	20 ± 3	25 ± 4
5	16 ± 0.5	20 ± 1	5	7 ± 4	9 ± 5
6	2 ± 0.1	2 ± 0	6	5 ± 5	7 ± 7
